# Antidepressant but Not Prophylactic Ketamine Administration Alters Calretinin and Calbindin Expression in the Ventral Hippocampus

**DOI:** 10.3389/fnmol.2018.00404

**Published:** 2018-11-06

**Authors:** Christina T. LaGamma, William W. Tang, Ashlea A. Morgan, Josephine Cecelia McGowan, Rebecca A. Brachman, Christine A. Denny

**Affiliations:** ^1^Division of Integrative Neuroscience, Research Foundation for Mental Hygiene, Inc. (RFMH)/New York State Psychiatric Institute (NYSPI), New York, NY, United States; ^2^Department of Psychiatry, Columbia University, New York, NY, United States; ^3^Doctoral Program in Neurobiology and Behavior, Columbia University, New York, NY, United States

**Keywords:** stress, social defeat, depression, resilience, dentate gyrus, hilus, doublecortin, neuronal marker

## Abstract

Ketamine has been found to have rapid, long-lasting antidepressant effects in treatment-resistant (TR) patients with major depressive disorder (MDD). Recently, we have also shown that ketamine acts as a prophylactic to protect against the development of stress-induced depressive-like behavior in mice, indicating that a preventative treatment against mental illness using ketamine is possible. While there is significant investigation into ketamine’s antidepressant mechanism of action, little is known about ketamine’s underlying prophylactic mechanism. More specifically, whether ketamine’s prophylactic action is molecularly similar to or divergent from its antidepressant action is entirely unknown. Here, we sought to characterize immunohistochemical signatures of cell populations governing ketamine’s antidepressant and prophylactic effects. 129S6/SvEv mice were treated with saline (Sal) or ketamine (K) either before a social defeat (SD) stressor as a prophylactic, or after SD as an antidepressant, then subsequently assessed for depressive-like behavior. Post-fixed brains were processed for doublecortin (DCX), calretinin (CR) and calbindin (CB) expression. The number of DCX^+^ neurons in the dentate gyrus (DG) of the hippocampus (HPC) was not affected by prophylactic or antidepressant ketamine treatment, while the number of CR^+^ neurons in the ventral hilus increased with antidepressant ketamine under SD conditions. Moreover, antidepressant, but not prophylactic ketamine administration significantly altered CR and CB expression in the ventral HPC (vHPC). These data show that while antidepressant ketamine treatment mediates some of its effects via adult hippocampal markers, prophylactic ketamine administration does not, at least in 129S6/SvEv mice. These data suggest that long-lasting behavioral effects of prophylactic ketamine are independent of hippocampal DCX, CR and CB expression in stress-susceptible mice.

## Introduction

Major depressive disorder (MDD) is one of the most prevalent and pervasive mental illnesses to date, affecting roughly 17% of the United States population (Kessler et al., 2005). The World Health Organization (WHO) asserts that depression is the leading cause of disability worldwide (World Health Organization, 2017). Yet, effective long-term treatments for MDD are still unavailable. Current treatment methods for depression include the use of selective serotonin reuptake inhibitors (SSRIs) which enhance maturation and synaptic integration of adult-born neurons (Wang et al., 2008). However, reduced efficacy and a delayed onset of action for such drugs are insufficient for treatment-resistant (TR) patients (Sackeim, 2001). Therefore, a faster and more targeted approach to treating MDD is needed.

Ketamine, an N-Methyl-D-aspartic acid (NMDA) receptor antagonist, has gained traction as a rapid-acting antidepressant. Since the 1960’s, ketamine has primarily been used as an anesthetic with potent analgesic properties (Mion and Villevieille, 2013). More recently, it has been shown that a single sub-anesthetic dose of ketamine can relieve existing symptoms in patients with TR depression in as little as 4 h, for up to 2 weeks (Berman et al., 2000). However, our lab discovered that ketamine can prevent the onset of depressive-like symptoms when administered prior to a stressor (Brachman et al., 2016), and attenuate learned fear in mice (McGowan et al., 2017). Thus, we have demonstrated that ketamine exerts protective effects against stress and enhances stress resilience. While many studies have explored the mechanism underlying ketamine’s rapid-acting antidepressant actions (Autry et al., 2011; Kavalali and Monteggia, 2012), little is known about the molecular pathways underlying ketamine’s prophylactic effects. Moreover, it is unclear whether the mechanism behind ketamine’s antidepressant effects are mechanistically similar or divergent from its prophylactic actions.

The hippocampus (HPC) is a region of particular interest for studying depression, as reductions in hippocampal size have been recorded in post-mortem studies of patients with MDD (Bremner et al., 2000). Volumetric decreases in the subgranular zone (SGZ) of the HPC, the dentate gyrus (DG), *cornu ammonis* 3 (CA3), as well as loss of granule cell (GC) number throughout the HPC of MDD patients may contribute to MDD etiology (Bremner et al., 2000; Boldrini et al., 2009). Studies have shown that SSRIs, as well as ketamine, increase the number of dividing cells in the SGZ of the HPC in both MDD patients, and rats, respectively (Boldrini et al., 2009; Soumier et al., 2016). Some antidepressant effects are abolished by ablating neurons in the DG of the HPC (Santarelli et al., 2003; Surget et al., 2008; Wang et al., 2008). Meanwhile, selectively increasing the number of adult born neurons in the HPC using a transgenic mouse model is sufficient to reduce anxiety-like and depressive-like behaviors in stressed mice (Hill et al., 2015). This suggests a critical role for the integrity of SGZ neurons in MDD pathogenesis.

Dysregulation of functional connectivity between subregions of the HPC and other limbic structures such as the medial prefrontal cortex (mPFC) may contribute to the development of MDD (Jacobs, 2002). Data reveal that input and output connections of the dorsal HPC (dHPC) and ventral HPC (vHPC) are distinct, suggesting that they play unique roles in affecting behavior (Swanson and Cowan, 1975). Disrupting hippocampal connectivity, synaptic plasticity and markers of hippocampal function have been shown to impact antidepressant action (Bremner et al., 2000; Carreno et al., 2016). Transient vHPC silencing, of vHPC to mPFC pathways, at the time of ketamine administration blocks the effects of ketamine, suggesting that ketamine may require intact vHPC to mPFC connections to be effective (Carreno et al., 2016). Moreover, our lab recently found evidence for the role of vHPC-mediated effects of prophylactic ketamine. Specifically, inhibition of ΔFosB, a transcription factor implicated in stress resilience, by viral expression of ΔJunD in ventral CA3 (vCA3) impairs the behavioral effects of prophylactic ketamine (Mastrodonato et al., 2018), suggesting the vHPC is necessary for eliciting a resilient phenotype following stress exposure. Taken together, these data show that there may be specific brain circuits dedicated to MDD pathogenesis and recovery, which could potentially be targeted with ketamine treatment.

Dysfunctional GABAergic networks between the PFC and HPC are thought to be responsible for the circuit-based explanation of MDD development (Croarkin et al., 2011; Luscher et al., 2011). Calretinin (CR), a GABAergic inhibitory marker of immature GCs and mossy cells, and calbindin (CB), another GABAergic inhibitory marker of mature GCs, are Ca^2+^ binding proteins on interneurons that modulate neuronal excitability in the HPC (Rüttimann et al., 2004; Camp and Wijesinghe, 2009). CR expression specifically is critical for the induction and maintenance of long-term potentiation (LTP) in the DG of mice (Gurden et al., 1998) and greater hippocampal network (Schurmans et al., 1997). Administration of a GABA receptor antagonist in CR deficient mice (CR^−/−^) restored LTP and synaptic transmission in the HPC, suggesting that expression of CR contributes to the control of synaptic plasticity by indirectly regulating GABAergic interneurons (Schurmans et al., 1997). Thus, we sought to determine whether dysregulation of homeostatic mechanisms in the HPC, as measured by changes in CR or CB expression, are implicated in ketamine’s prophylactic effects.

We sought to investigate whether CR, CB, or doublecortin (DCX), a marker of proliferating neurons, were altered following prophylactic or antidepressant ketamine administration. These three markers were selected to determine whether proliferation of immature neurons or activity of inhibitory neurons mediating synaptic integration and GC maturation in the HPC are implicated in the behavioral efficacy of ketamine following a social defeat (SD) stressor. The goal was to characterize immunohistochemical markers following prophylactic or rapid-acting antidepressant ketamine administration and identify ketamine-dependent changes in the cellular profile of hippocampal neurons. A comparative investigation into the neurochemical origins governing ketamine’s bifold behavioral effects may advance targeted development of ketamine-driven interventions for psychiatric illnesses.

## Materials and Methods

### Mice

Male 129S6/SvEvTac mice were purchased from Taconic (Hudson, NY, USA) at 7 weeks of age. Mice were housed 4–5 per cage in a 12-h (06:00–18:00) light-dark colony room at 22°C. Food and water were provided *ad libitum*. Behavioral testing was performed during the light phase. All experiments were approved by the Institutional Animal Care and Use Committee (IACUC) at the New York State Psychiatric Institute (NYSPI).

### Behavioral Assays

Mice were administered SD, the forced swim test (FST), dominant interaction (DI), novelty suppressed feeding (NSF), elevated plus maze (EPM) and contextual fear conditioning (CFC) as previously defined (Brachman et al., 2016). Brain tissue from this current study was processed from the SD cohorts published in Brachman et al. (2016).

### Drugs

A single injection of saline (0.9% NaCl) or ketamine (30 mg/kg; Ketaset III, Ketamine HCl, Fort Dodge Animal Health, Fort Dodge, IA, USA) was administered once during the course of each experiment. Ketamine was prepared in physiological saline and all injections were administered intraperitoneally (i.p.) in volumes of 0.1 cc per 10 mg body weight.

Thirty mg/kg was chosen as a result of the dose titration experiment performed in Brachman et al. (2016), which determined that a single injection of 30 mg/kg of ketamine, but not 10 mg/kg or 90 mg/kg, administered 1 week prior to SD, blocked the onset of depressive-like symptoms in 129S6/SvEv male mice.

### Sample Preparation

Mice were deeply anesthetized with ketamine (100 mg/kg) and xylazine (10 mg/kg) and transcardially perfused with 0.1 M phosphate buffer saline (1× PBS), followed by cold 4% paraformaldehyde (PFA)/1× PBS. Brains were postfixed in PFA overnight in 4% PFA, then cryoprotected in 30% sucrose/1× PBS for 3 days at 4°C. Serial coronal sections (35 μm) were cut through the entire HPC using a cryostat, then stored in 1× PBS with 0.1% sodium azide.

### Immunohistochemistry

DCX immunohistochemistry was performed as previously described (Denny et al., 2012).

For CR and CB immunohistochemistry, sections were rinsed three times in 1× PBS with 0.5% Triton X-100 (PBST) at room temperature (RT) for 10 min each. Sections were quenched in 3% H_2_O_2_ in PBST with MeOH (1:1) for 15 min, rinsed three more times in PBST for 10 min each, then blocked with 10% normal donkey serum (NDS) in PBST (blocking solution) for 2 h at RT. Incubation with primary antibody was performed at 4°C overnight (goat anti-CR, 1:1,000, Millipore, AB1550; mouse anti-CB, 1:5,000, Swant, CB300) in blocking solution. Sections were then washed in 1× PBS three times for 10 min each and incubated in secondary antibody (donkey anti-goat and donkey anti-rabbit, 1:500, Jackson Immuno Research, West Grove, PA, USA) for 2 h at RT. All sections were washed three times with 1× PBS, mounted on glass slides, and coverslipped with ProLong Gold (Invitrogen, Carlsbad, CA, USA).

Tissue from prophylactic and antidepressant treatment groups were processed separately.

### Cell Quantification and Image Acquisition

DCX^+^ and CR^+^ cells were counted on a Zeiss Axio Observer A1 upright microscope. All sections along the dorsoventral axis for the entire DG and hilus were counted. Dorsal, intermediate and ventral counts were averaged across respective groups.

### Confocal Microscopy

CR and CB imaging was performed using a Leica TCS SP8 multiphoton microscope with LAS X software (v. 1.1.0.12420). Identification of positively labeled CR and CB expressing regions in the HPC involved acquiring three dorsal, intermediate and ventral sections per mouse brain slice at 20×. All individual panels were acquired at a thickness of 1.4 μm. Split panel and z-stack analysis was performed using the LAS X image browser to determine expression of CR and CB. Representative expression levels of CR and CB were compared across all sections using identical exposure conditions. ImageJ^1^ software was used to outline regions for fluorescent intensity quantification and averaged across respective groups.

### Statistical Analysis

All data were analyzed using StatView 5.0 software (SAS Institute, Cary, NC, USA) or Prism 5.0a (Graphpad Software, Inc., La Jolla, CA, USA). Alpha was set to 0.05 for all analyses. The effect of Drug or Group was analyzed using an analysis of variance (ANOVA). Significant ANOVAs were followed with unpaired *t*-tests where appropriate. All statistical tests and *p* values are listed in Supplementary Table S1.

## Results

### Prophylactic but Not Antidepressant Ketamine Administration Attenuates Stress-Induced Depressive-Like Behavior

**Figure 1:**
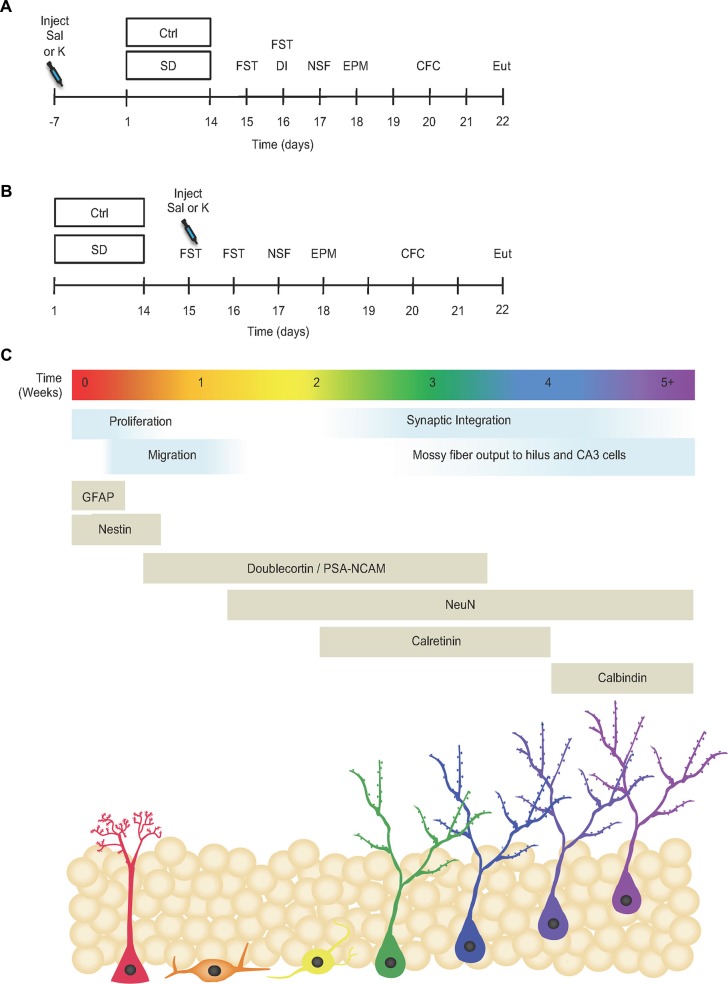
Prophylactic but not antidepressant ketamine administration attenuates stress-induced depressive-like behavior. **(A)** Prophylactic and **(B)** antidepressant experimental timelines (adapted from Brachman et al., 2016). **(C)** Timeline indicating the developmental stages and neuronal expression markers throughout adult-hippocampal neurogenesis (adapted from von Bohlen und Halbach, 2007; Duan et al., 2008; Denny et al., 2012). Ctrl, control; SD, social defeat; Sal, saline; K, ketamine; FST, forced swim test; DI, dominant interaction; NSF, novelty suppressed feeding; EPM, elevated plus maze; CFC, contextual fear conditioning; Eut; euthanize; GFAP, glial fibrillary acidic protein; PSA-NCAM, polysialylated-neural cell adhesion molecule; NeuN, neuronal nuclei; CA3, *cornu ammonis* III.

In the first set of experiments, mice were administered a single injection of either saline or ketamine (30 mg/kg) 1 week before SD (Figure 1A). Mice were then administered behavioral tests to assay depressive-like, anxiety-like and cognitive behavior. We previously reported that prophylactic ketamine prevented stress-induced depressive-like behavior, but not anxiety-like behavior (Figures 1, 2 in Brachman et al., 2016).

In a second set of experiments, mice underwent SD for 2 weeks and then were administered saline or ketamine (30 mg/kg) 1 day later (Figure 1B). Mice again underwent a number of behavioral tests. We previously reported that prophylactic, but not antidepressant, ketamine improved stress-induced depressive-like behavior in SD mice (Figure 5 in Brachman et al., 2016). Here, brain tissue from these cohorts were processed for hippocampal markers (Figure 1C).

### Neither Prophylactic Nor Antidepressant Ketamine Administration Alters DCX^+^ Cells Throughout the DG

Previously, we have shown that neither ketamine nor a SD stressor alters the number of Ki67^+^ cells in the HPC of male 129S6/SvEv mice (Brachman et al., 2016). Ki67 is nuclear protein that is expressed in dividing cells, throughout the cell’s entire mitotic process (Scholzen and Gerdes, 2000). Given that ketamine had no significant effect on the number of Ki67^+^ cells in the HPC, we next quantified the effect of a higher dosage of ketamine on another principle marker for mitotic cells division in the rodent brain, 5-Bromo-2′-deoxyuridine (BrdU; Gratzner, 1982; Kee et al., 2002). The number of BrdU^+^ cells increased with higher doses of ketamine when compared with administration of saline or pentobarbital (PB; Supplementary Figure S1), indicating that higher, but not lower doses of ketamine are effective at increasing proliferation.

**Figure 2:**
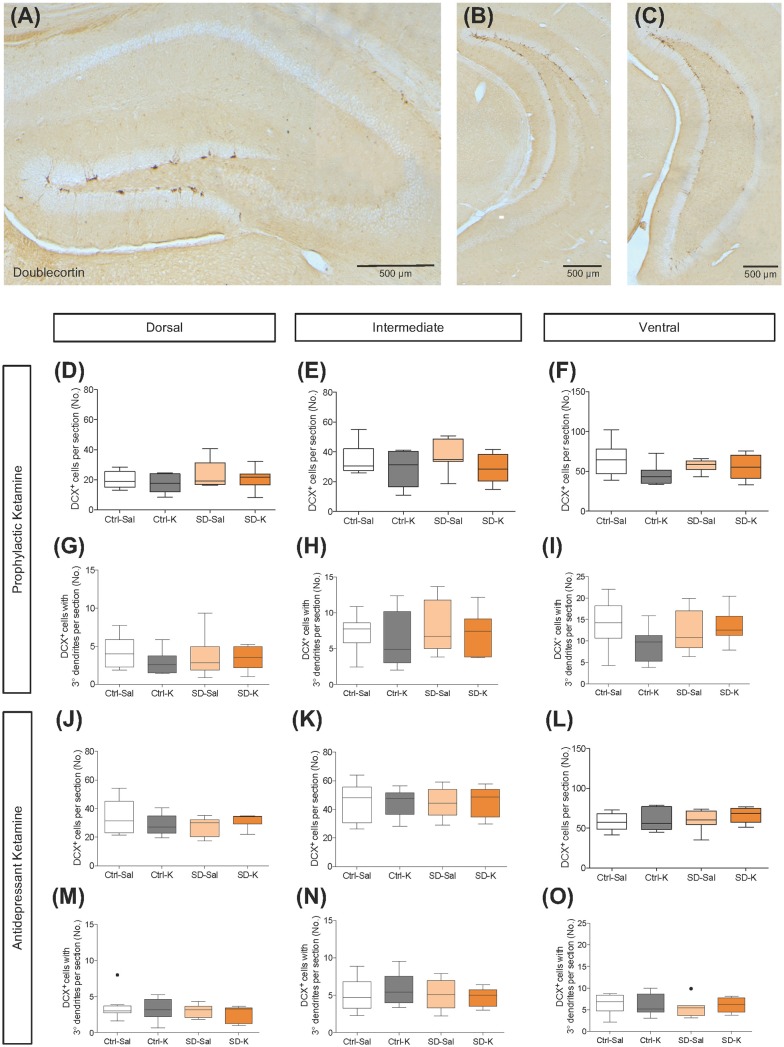
Neither prophylactic nor antidepressant ketamine administration alters DCX expression throughout the DG. **(A–C)** DCX expression in the DG along the dorsoventral axis of the hippocampus (HPC). Prophylactic ketamine administration does not alter the number of **(D–F)** DCX^+^ cells or **(G–I)** DCX^+^ cells with tertiary dendrites in the dorsal, intermediate, or ventral DG. Antidepressant ketamine administration does not alter the number of **(J–L)** DCX^+^ cells or **(M–O)** DCX^+^ cells with tertiary dendrites in the dorsal, intermediate, or ventral DG. There was a significant effect of Drug in the dorsal DG for prophylactic and antidepressant treatment groups. Black dots in **(M)** and **(O)** represent outliers. Scale bars represent 500 μm. (*n* = 6–9 male mice per group). Error bars represent ± SEM. Ctrl, control; SD, social defeat; Sal, saline; K, ketamine; DCX, doublecortin; DG; dentate gyrus.

Since the increase in expression of BrdU cells may not be visible at lower doses in the 129S6/SvEv strain, as they exhibit low levels of neurogenesis, we chose to look at post-division DCX expressing cells. DCX is expressed by determined progenitor cells (Type 2b/3) in the SGZ (Filippov et al., 2003) and is considered a marker of immature neurons (Brown et al., 2003; Rao and Shetty, 2004). To determine whether immature hippocampal neurons are implicated in the prophylactic or antidepressant actions of ketamine, we first quantified the number of DCX^+^ cells per section in the DG along the dorsoventral axis (Figures 2A–C). For prophylactic ketamine administration, there was no effect of Group (control (Ctrl) vs. SD) or Drug (saline (Sal) vs. ketamine (K)) on the number of DCX^+^ cells per section in the intermediate and ventral DG (Figures 2E,F), but there was an effect of Drug in the dorsal DG (*p* = 0.0331; Figure 2D). There was no effect of Group or Drug on DCX^+^ cells with tertiary dendrites throughout the DG (Figures 2G–I). However, the number of DCX^+^ cells (Supplementary Figure S2A) and DCX^+^ cells with tertiary dendrites (Supplementary Figure S2B) increased along the dorsoventral axis (ANOVAs for region, *p* < 0.0001).

In mice that were administered a single injection of ketamine as a rapid-acting antidepressant after SD, there was an effect of Drug in the dorsal DG (*p* = 0.0460; Figure 2J). However, the number of DCX^+^ cells per section in the intermediate and ventral DG were not significantly influenced by either Group or Drug (Figures 2K,L). DCX^+^ cells with tertiary dendrites were not significantly affected by Group or Drug (Figures 2M–O). The black dot in Figure 2O represents an outlier. Once again, the total number of DCX^+^ cells as well as the total number of DCX^+^ cells with tertiary dendrites increased along the dorsoventral axis (Supplementary Figures S2C,D; ANOVAs for region, *p* < 0.0001). These data suggest that the DCX expressing immature neurons are not implicated in prophylactic or antidepressant ketamine treatment in 129S6/SvEv mice.

### Antidepressant Ketamine Administration Alters CR^+^ Expression in the HPC

**Figure 3:**
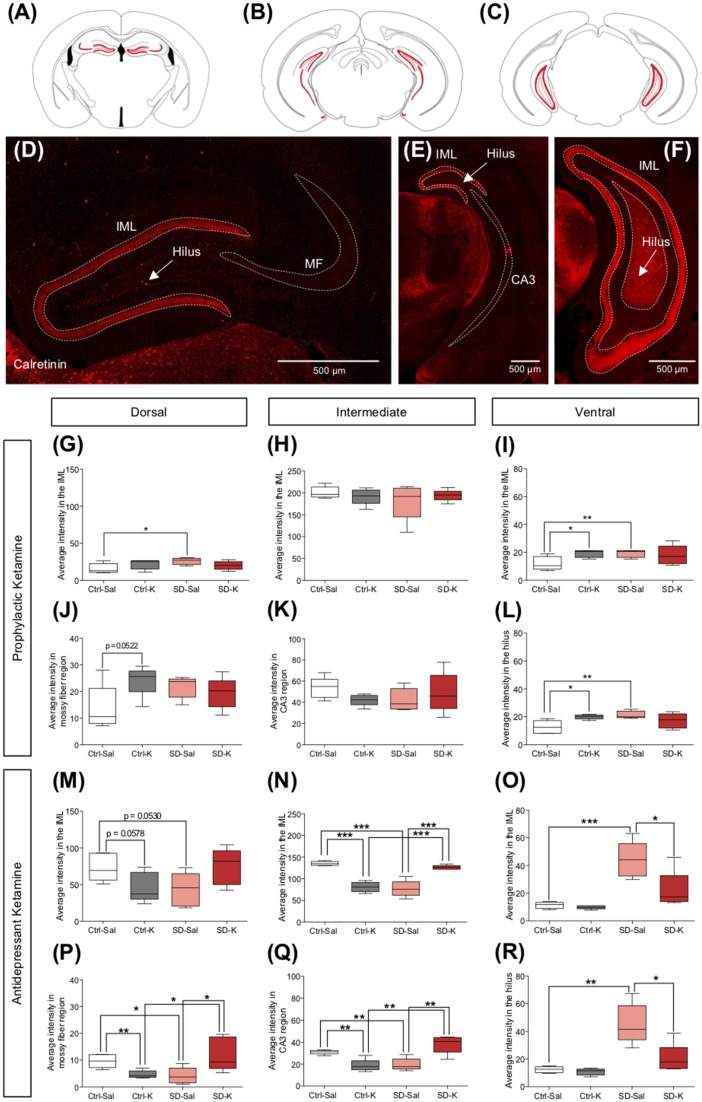
Antidepressant ketamine administration alters CR expression in the HPC. **(A–C)** Representative atlas images of positively labeled CR expression in the inner molecular layer (IML) of the dorsal, intermediate and ventral HPC (vHPC), dorsal mossy fiber (MF) region, intermediate CA3, and ventral hilus. **(D–F)** Representative images indicating CR fluorescent expression throughout the HPC. Arrows indicate CR^+^ cells along the dorsoventral axis of the hilus region. **(G,H)** Prophylactic ketamine does not alter CR expression in the dorsal or intermediate IML. **(I)** In the ventral hilus, ketamine significantly increases CR expression under Ctrl conditions. **(J,K)** Neither prophylactic ketamine nor SD alters CR expression in the dorsal MF region and intermediate CA3. **(L)** In the ventral MF region, ketamine significantly increases CR expression in the Ctrl mice. **(M)** Neither antidepressant ketamine nor SD alters CR expression in the dorsal IML. **(N)** In the intermediate IML, ketamine significantly decreases CR expression in the Ctrl mice, whereas ketamine significantly increases CR expression in the SD mice. **(O)** In the ventral IML, ketamine significantly decreases CR expression in SD mice. **(P,Q)** In the dorsal and intermediate HPC, ketamine significantly decreases CR expression in the MF region for in Ctrl mice, whereas ketamine significantly increases CR expression in the SD mice. **(R)** In the ventral hilus, ketamine significantly reduces CR expression in the SD mice. (*n* = 5 male mice per group). Error bars represent ± SEM. *p < 0.05, **p < 0.01, ***p < 0.001. Ctrl, control; SD, social defeat; Sal, saline; K, ketamine; CR, calretinin.

We next investigated the Ca^2+^ binding protein CR, which is expressed in axonal arborizations of the inner molecular layer (IML) along the dorsoventral axis of the DG (Volz et al., 2011). Here, we sought to explore changes in IML throughout the dorsoventral axis, as well as mossy fiber (MF) region, CA3 and hilus in the dorsal, intermediate and vHPC respectively, as indicated in the schematic diagrams (Figures 3A–C). Representative dorsal, intermediate and ventral images reveal CR expression throughout the HPC (Figures 3D–F).

Following prophylactic ketamine administration, there was no effect of Group or Drug on CR expression in the dorsal IML, though the interaction did reach significance; SD-Sal mice had significantly higher CR expression than Ctrl-Sal mice (Figure 3G). There was no effect of Group, Drug, or the interaction in the intermediate IML (Figure 3H). In the ventral IML, there was no effect of Group or Drug for mice treated with prophylactic ketamine, but there was a significant interaction (Figure 3I). Ctrl-K and SD-Sal mice had significantly higher CR expression than Ctrl-Sal mice. In the MF and CA3 region, there was no significant effect of Group or Drug in either the dorsal or intermediate HPC (Figures 3J,K). However, in the vHPC, the interaction reached significance (Figure 3L). Ctrl-K and SD-Sal mice had significantly higher CR expression in the hilus than Ctrl-Sal mice. These data indicate that prophylactic ketamine administration does not significantly influence expression of CR axonal projections in the HPC.

In the antidepressant ketamine-treated group, the was no significant effect of Group or Drug on CR expression in the dorsal or intermediate IML, though there was a significant interaction in both regions (Figures 3M,N). In the dorsal IML, no subsequent tests reached significance (Figure 3M). However, in the intermediate IML, all follow up tests reached significance (Figure 3N). Ctrl-K mice had significantly less CR expression in the IML when compared with Ctrl-Sal mice. SD-K mice had significantly more CR expression in the IML when compared with SD-Sal mice. In the ventral IML, there was a significant effect of Group, Drug and the interaction (Figure 3O). While SD-Sal mice had increased expression when compared with Ctrl-Sal mice, SD-K mice had reduced expression when compared to Ctrl-Sal mice. In the MF region, there was no significant effect of Group or Drug but there was a significant interaction in both the dorsal and intermediate MFs (Figures 3P,Q). All comparisons reached significance. Lastly, in the vHPC, there was a significant effect of Group, Drug and the interaction on CR expression in the ventral hilus (Figure 3R). While SD-Sal mice had increased expression when compared with Ctrl-Sal mice, SD-K mice had reduced expression to that of Ctrl-Sal mice. These data indicate the antidepressant ketamine has opposing effects on CR expression in the HPC depending on the absence or presence of an SD stressor.

### Antidepressant Ketamine Administration Significantly Increases the Number of CR^+^ Cells in the Ventral Hilus

**Figure 4:**
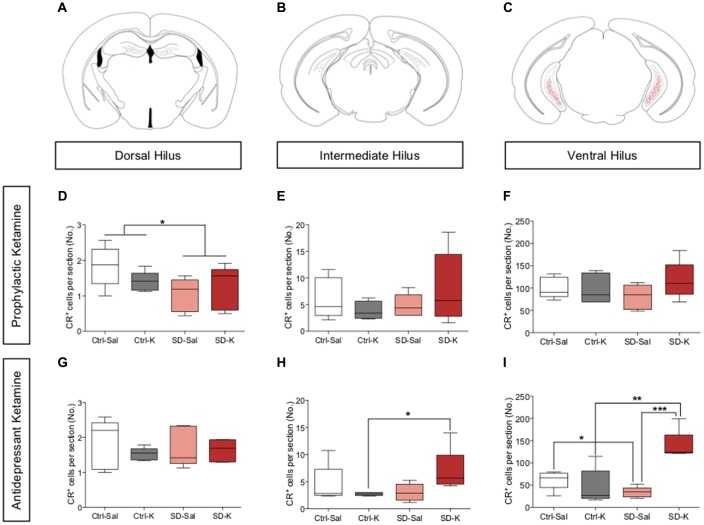
Antidepressant ketamine administration significantly increases the number of CR^+^ cells in the ventral hilus. **(A–C)** Representative atlas images of positively labeled CR^+^ cells in the dorsal, intermediate and ventral hilus. **(D–F)** Prophylactic ketamine administration has no effect on the number of CR^+^ cells in the dorsal, intermediate, or ventral hilus. **(G,H)** Antidepressant ketamine administration does not alter the number of CR^+^ cells in the dorsal or intermediate hilus. **(I)** Ketamine significantly increases the number of CR^+^ cells in the ventral hilus of SD mice. (*n* = 5 male mice per group). Error bars represent ± SEM. *p < 0.05, **p < 0.01, ***p < 0.001. Ctrl, control; SD, social defeat; Sal, saline; K, ketamine; IML, inner molecular layer; MF, mossy fiber; CA3, *cornu ammonis* III; CR, calretinin.

Given the significant changes we saw in hippocampal CR expression following ketamine administration, we next counted the CR^+^ cell bodies located throughout the hilus of the HPC. In addition to labeling arborizations in the IML, CR is expressed in hilar mossy cells, which are strongly implicated in synaptic integration (Fujise et al., 1998; Gurden et al., 1998; Fujise and Kosaka, 1999; Seress et al., 2008; Scharfman, 2016). CR^+^ cells in the SGZ and hilus region were then counted along the dorsoventral axis of the HPC (Figures 4A–C).

For mice treated with prophylactic ketamine, there was a significant effect of Group, but not of Drug in the dorsal hilus (Figure 4D). SD mice had significantly fewer CR^+^ cells when compared with Ctrl mice. However, there was no effect of either Group or Drug in the intermediate or ventral hilus of prophylactic ketamine-treated mice (Figures 4E,F). These data indicate that prophylactic ketamine does not significantly impact the number of CR^+^ cells in the hilus.

For mice treated with antidepressant ketamine, there was no significant effect of Group or Drug in the dorsal or intermediate hilus (Figures 4G,H). However, the interaction did reach significance in the intermediate hilus. SD-K mice had significantly more CR^+^ cells when compared with Ctrl-K mice (Figure 4H). In the ventral hilus, there was a significant effect of Group, Drug and a significant interaction (Figure 4I). SD-K mice had significantly more CR^+^ cells when compared with the Ctrl-K and SD-Sal groups. SD-Sal mice had significantly fewer CR^+^ cells when compared with Ctrl-Sal. These data suggest that antidepressant ketamine treatment preferentially targets ventral CR^+^ cells.

### Antidepressant Ketamine Administration Alters CB^+^ Expression in the HPC

**Figure 5:**
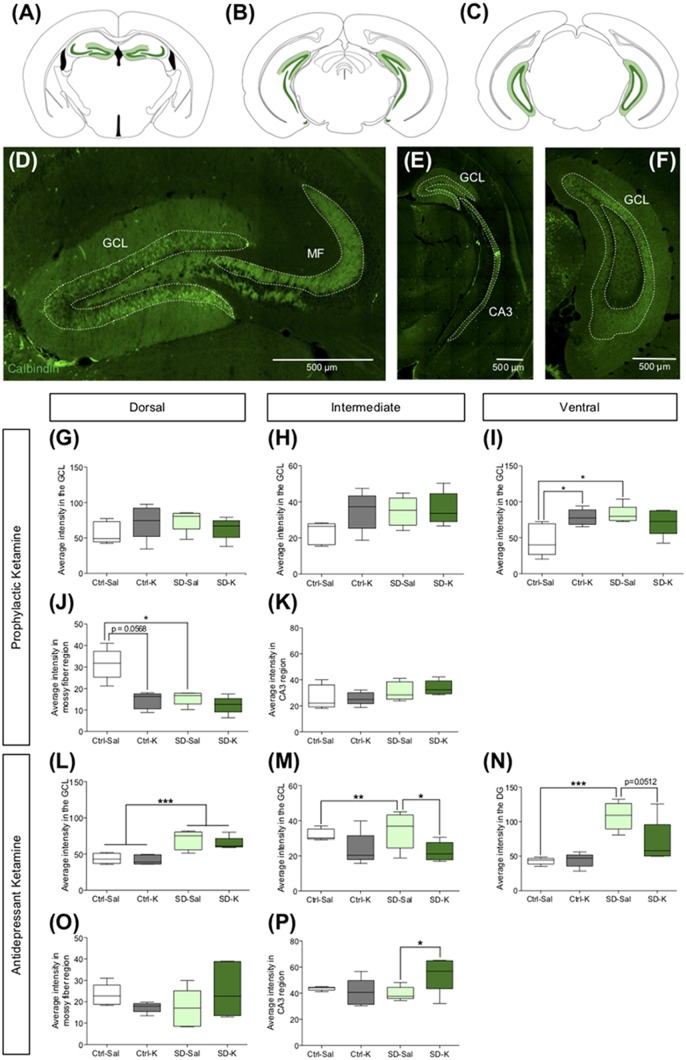
Antidepressant ketamine administration alters CB expression in the HPC. **(A–C)** Representative atlas images of CB^+^ fluorescence in the GCL of the dorsal, intermediate and vHPC, dorsal MF, and intermediate CA3. **(D–F)** Representative images indicating CB fluorescent expression throughout the HPC. **(G,H)** In the dorsal and intermediate GCL, neither ketamine nor SD alters CB expression. **(I)** In the ventral DG, ketamine significantly increases CB expression in the Ctrl mice. **(J,K)** Prophylactic ketamine administration has no effect on MF CB expression in the dorsal and intermediate HPC. **(L)** Antidepressant ketamine administration has no effect on CB expression in the dorsal GCL. **(M)** In the intermediate GCL, ketamine significantly decreased CB expression under SD conditions. **(N)** In the ventral GCL, antidepressant ketamine administration has no effect on CB expression. **(O,P)** Neither antidepressant ketamine nor SD alters CB expression in the dorsal MF region. In the intermediate MF region, ketamine significantly increases CB expression in SD mice. (*n* = 5 male mice per group). Error bars represent ± SEM. *p < 0.05, **p < 0.01, ***p < 0.001. Ctrl, control; SD, social defeat; Sal, saline; K, ketamine; GCL, granule cell layer; MF, mossy fiber; CA3, *cornu ammonis* III; CB, calbindin.

Next, we explored whether ketamine administration alters CB expression in the HPC. CB, a Ca^2+^ binding protein that is primarily expressed in mature GCs (Schwaller, 2010; Todkar et al., 2012), was assessed throughout the dorsoventral axis (Figures 5A–C). Representative atlas images indicate CB expression in the GC layer (GCL) of the DG, MF terminals and CA3 (Figures 5D–F). Following prophylactic administration, there was no impact of Group or Drug on CB expression in the GCL of the dorsal, intermediate, or ventral DG (Figures 5G–I). However, there was a significant interaction in the ventral GCL (Figure 5I); Ctrl-K and SD-Sal mice had significantly higher CB expression than Ctrl-Sal mice. In the dorsal MF region, although there was no impact of Group or Drug, the interaction did reach significance (Figure 5J). SD-Sal mice had significantly less CB expression when compared with Ctrl-Sal mice. In the intermediate HPC, there was also no significant impact of Group or Drug (Figure 5K). These data indicate that prophylactic ketamine exerts its protective effect without modulating CB expression.

Following antidepressant administration, there was no impact of Drug on CB expression in the dorsal GCL of the DG. However, mice that underwent SD had significantly higher CB expression than Ctrl mice (Figure 5L). In the intermediate GCL, while there was no effect of Group or Drug, there was a significant interaction (Figure 5M). SD-Sal mice had significantly more CB expression when compared with SD-K and Ctrl-Sal mice. In the ventral GCL, there was a significant effect of Group as well as Interaction (Figure 5N). SD mice had increased CB expression when compared with Ctrl mice. Specifically, SD-Sal mice exhibited more CB expression when compared with Ctrl-Sal mice. In the dorsal MF region, there was no effect of Group or Drug (Figure 5O). However, in the intermediate HPC there was a significant interaction in the MF region (Figure 5P). SD-K mice had increased CB expression in the MFs when compared with SD-Sal mice. These data indicate that antidepressant ketamine has a modest impact on CB expression in the HPC.

## Discussion

In this study, we found that antidepressant, but not prophylactic, ketamine administration significantly altered CR and CB expression in the vHPC, while DCX expression was not affected by either treatment. These data suggest that while antidepressant ketamine actions may be mediated by CR and CB expressing cells, prophylactic ketamine administration is not, at least in male 129S6/SvEv mice. To our knowledge, this is the first study to compare the expression profile of neuronal markers in the HPC between prophylactic and antidepressant treatment paradigms.

Prior to this study, the extent to which DCX expressing neurons in the DG contribute to ketamine’s prophylactic and antidepressant effects was not thoroughly investigated. In our initial publication, we analyzed DCX expression but did not specifically consider DCX^+^ cells with tertiary dendrites or investigate DCX expression throughout the HPC (Brachman et al., 2016). A primary incentive for further focusing on DCX expression was due to the observation that pharmacological ablation of DCX-expressing cells in the HPC of glial fibrillary acidic protein (GFAP)–thymidine kinase (TK) mice demonstrated a blunted stress response and increased depressive-like behavior (Snyder et al., 2011). Moreover, while new hippocampal neurons are not required for the acute antidepressant effects of ketamine in rats, it remained unknown whether they were required for prophylactic ketamine (Soumier et al., 2016). Thus, it was necessary to further explore whether DCX was implicated in ketamine’s antidepressant and prophylactic effects in mice.

We found that administration of a single dose of ketamine either before or after SD does not have long term effects on the number of DCX^+^ cells throughout the DG nor those with tertiary dendrites. These results corroborated evidence in our earlier article showing that neither the number of Ki67^+^ cells nor the number of DCX^+^ cells changed with SD or ketamine treatment (Brachman et al., 2016). Additionally, our data aligned with a previous study showing that a single injection of (*S*)-ketamine does not alter the number of DCX^+^ cells in rats (Soumier et al., 2016). Taken together however, these results contrast work by Ma et al. (2013), demonstrating that the number of DCX^+^ cells increased in the HPC 1 week after a single administration of ketamine (7 mg/kg). These data suggest that there is a specific time window during which DCX expression should be analyzed. It is possible that in our experimental timeline, the duration between ketamine administration and immunohistochemical analysis was too long for significant changes in DCX expression to be visible. Thus, it remains to be determined, to what extent DCX plays a role in ketamine’s actions.

The mouse strain utilized in our study should also be considered when discussing our findings. Most experiments studying the role of ketamine previously utilized C57Bl/6J mice, a more resilient strain (Clark et al., 2011). We chose to use 129S6/SvEv mice because they are more susceptible to stress than C57Bl/6J mice, but they have significantly lower baseline levels of neurogenesis (Kempermann and Gage, 2002; van Bogaert et al., 2006). Moreover, in depression studies, C57B1/6J mice typically have to be stressed with a chronic corticosterone paradigm in order to see relevant behavioral changes in stress levels (David et al., 2009). Thus, in using 129S6/SvEv, it is conceivable that at a lower baseline level, a single dose of ketamine was not strong enough to substantially change DCX expression.

We specifically chose not to investigate BrdU expression as a marker for cellular proliferation in the SD study because of its ability to impact cell fate (Duque and Rakic, 2011), thus, serving as a possible confound in determining solely ketamine’s effect on stress. Furthermore, there is evidence suggesting that BrdU can have unintended generative effects on neurogenesis (Kee et al., 2002), as well as potential negative side-effects including the production of mutated cells with subsequent tissue abnormalities in the developing mouse (Kolb et al., 1999). There is uncertainty in the success of BrdU diffusion across the blood brain barrier (Cameron and McKay, 2001), which raises doubt on its effective incorporation into the DNA of diving cells. Taken together, we thought it was sufficient to demonstrate that at least high doses of ketamine have the potential to stimulate rapid proliferation of newborn cells.

Our rationale for investigating CR and CB next was threefold. First, these markers are expressed at discrete timepoints in a neuron’s lifespan. CR is expressed from 2 weeks to 4 weeks of age and CB is expressed from 4 weeks of age and beyond, without overlapping in expression profiles (Ming and Song, 2005; von Bohlen und Halbach, 2007; Duan et al., 2008; Christian et al., 2014), thus, permitting for timestamp experiments. Second, CR and CB are Ca^2+^-binding proteins that are implicated in neuronal excitability, Ca^2+^ buffering, and the induction of LTP (Meltzer et al., 2005; Camp and Wijesinghe, 2009). It is possible that circuit-level Ca^2+^ homeostasis mediates ketamine’s behavioral effects. Third, Kobayashi et al. (2010) found that chronic fluoxetine administration decreased expression of CB, but increased expression of CR, demonstrating a dematuration effect and a potential new mechanism for antidepressant action. Lastly, we performed an immunohistochemical analysis of these markers including DCX and BrdU because there exists a large body of literature which uses immunohistochemistry to successfully plot the time course of neuronal differentiation and maturation in the HPC (Brandt et al., 2003; Ming and Song, 2005; von Bohlen und Halbach, 2007). Moreover, immunohistochemistry allows for both phenotypic and quantitative analysis of the desired sections. Therefore, using this method, we sought to investigate whether prophylactic and/or antidepressant treatment may also differentially alter CB and CR expression.

Before further assessing the roles of CR and CB expression in ketamine’s effects, however, we considered the differential roles of dorsal, intermediate and vHPC regions. Until relatively recently, subregion-specific analyses of the HPC along the dorsoventral axis have largely been ignored. Bannerman et al. (2014) showed that the dHPC is implicated in spatial cognition, whereas the vHPC is implicated in mood and stress-related behavior. Fanselow and Dong (2010) noted that some literature treats the HPC as purely a cognitive structure involved in learning and memory, whereas others treat the HPC as a principle regulator of emotional dysfunction and psychopathology. Here, we performed an analysis of hippocampal subregions along the dorsoventral axis. Many of the expression changes were subregion specific (e.g., in the vHPC, but not in the dHPC), suggesting that the HPC should not be treated as a homogenous structure. Despite this, a limitation of this study is that we did not investigate other brain regions implicated in depressive-like behavior alongside the HPC, such as the PFC and nucleus accumbens (Teyssier et al., 2011; Bagot et al., 2015). In future studies, it would be valuable to expand our investigation to include additional brain circuitry implicated in MDD.

We report a significant increase in both DCX and CR cell counts along the dorsoventral axis of the HPC. These results contrast with one study demonstrating a reduction in DCX^+^ and CR^+^ cells in the vHPC (Jinno, 2011). However, our CR results align with those of Fujise et al. (1998), showing that the number of CR^+^ mossy cells in the vHPC is greater at baseline per area than in the dHPC. This discrepancy could easily be explained by the differences in tissue processing, as we processed coronal sections which include a dorsal and ventral component as opposed to other processing methods (e.g., sagittal sections). The apparent structural variations throughout the HPC though further suggests differential roles in the pathophysiology of HPC-dependent mood disorders.

Upon analyzing CR and CB expression, we found that antidepressant ketamine had opposing effects on CR expression between Ctrl and SD groups. In dorsal and intermediate regions of the HPC, ketamine decreased CR expression in Ctrl mice, but increased CR expression in SD mice. These effects were not mimicked in CB expressing subregions following antidepressant ketamine treatment. While the opposing effects of ketamine under Ctrl and SD conditions are unique, they suggest that stress may render cells functionally different in response to pharmacological or environmental interventions.

Following prophylactic administration, ketamine had minimal effects on CR and CB expression. Interestingly, CR expression was low in the dHPC and vHPC, but highest in the intermediate subregion of the HPC. Conversely, CB expression was high in the dHPC and vHPC, but lowest in the intermediate subregion of the HPC. This data could support findings of increased CR immunoreactivity in regions of decreased CB immunoreactivity in the HPC (Kobayashi et al., 2010). Further investigation is needed to determine the functional relevance of why CR expression is highest in the intermediate area of the HPC, as this phenomenon is largely unexplored.

It is worth noting that although we found significant molecular changes in CR and CB expression for the antidepressant treatment groups, in our previous article, these mice had no significant changes in depressive-like behavior in the FST. Conversely, here, we report no significant changes in DCX, CR, or CB expression for prophylactic treatment groups, yet it was previously found that prophylactic ketamine to SD mice resulted in reduced immobility time in the FST (Brachman et al., 2016). The timepoint at which expression was measured may have been too late to see changes in neuronal proliferation relative to ketamine injection, regardless of the paradigm. DCX is expressed 7 days after a neuron is born and starts to disappear after 3 weeks (Ge et al., 2008; Christian et al., 2014). Given that mice were sacrificed 21 days after the injection in the prophylactic experiment, it is possible that there was a transient change in these markers’ expression and if mice were sacrificed more proximal to the injection, these changes may have been visible. While the behavioral changes in Brachman et al. (2016) do not corroborate the molecular changes in this study, it is likely that CR and CB are not the correct markers to explain the differential behavioral effects of ketamine.

Overall, these data suggest that CR and CB expression may be critical in mediating some of ketamine’s antidepressant actions, and that long-lasting changes in neuronal expression of DCX, CB and CR may not explain prophylactic efficacy of ketamine. These findings begin to elucidate some of the molecular-based changes associated with ketamine treatment and provide a panel of markers in two ketamine-treated groups. Therefore, we believe these results narrow down the possible targets of ketamine treatment and will inform future studies investigating ketamine as a prophylactic and/or as a rapid-acting antidepressant.

## Author Contributions

CD oversaw the entire project as principal investigator. CL wrote the manuscript and performed the majority of the experimental work. WT, AM, JM and RB ran experiments, performed image analysis and helped write/edit portions of the manuscript.

## Conflict of Interest Statement

CL, WT and AM reported no biomedical financial interests or potential conflict of interests. RB, JM and CD are named on a non-provisional patent application for the prophylactic use of ketamine against stress-related psychiatric disorders.

## Footnotes

^1^https://imagej.nih.gov/ij/
