# Supplementary material for: Antidepressant but Not Prophylactic Ketamine Administration Alters Calretinin and Calbindin Expression in the Ventral Hippocampus
Source: Front Mol Neurosci. 2018 Nov 6;11:404. doi: 10.3389/fnmol.2018.00404 (PMC6232342; doi:10.3389/fnmol.2018.00404)
Supplement: Supplementary file 2 [file Data_Sheet_1.pdf]

SUPPLEMENTARY INFORMATION

Antidepressant but not prophylactic ketamine administration alters calretinin and calbindin expression in the ventral hippocampus

Authors

Christina T. LaGamma[1], William W. Tang[2], Ashlea A. Morgan[3], Josephine C. McGowan[3], Rebecca A. Brachman[2], and Christine A. Denny[1,2,*]

Methods

Social Defeat (SD). One adult male aggressor CD-1 mouse was single-housed in a Macrolon® polycarbonate resin cage (15.25 in x 7.8 in x 9.5 in) (Animal Care Systems, Inc., Centennial, CO). A male 129S6/SvEvTac mice (9 weeks of age) was placed on the adjacent side of the CD-1 mouse's cage, separated by a stainless-steel partition (Animal Care Systems, Inc., Centennial, CO, P/N C79171). Once a day for 2 weeks, the barrier between the mice was removed and 3 antagonistic encounters were allowed to occur between the CD-1 mouse and the intruder 129S6/SvEvTac mouse. Each encounter lasted approximately 5 minutes, after which the mice were separated by the partition once again. This allowed for olfactory and auditory communication but limited visual or tactile contact. This procedure consistently yielded a submissive phenotype in the experimental intruder mice. Control (Ctrl) mice were group housed 4 to 5 per cage in Macrolon® polycarbonate resin cages without dividers.

Dominant Interaction (DI). Dominant interaction was performed as previously described in Donahue et al., 2014. Mice were briefly placed into a large open field that 2 upside-down wire mesh pencil cups. One of the pencil cups served as a novel stimuli. The other inverted pencil cup contained an aggressor CD-1 mouse. The mice were placed into the middle of a bright arena and were allowed to explore for 10 minutes. All behavioral sessions were videotaped, but only the first 5 minutes of each video were later analyzed using behavioral tracking software (TopScan, CleverSys, Reston, VA).

Forced Swim Test (FST). The FST is a reliable paradigm to test depressive-like behavior in rodent models of depression. We administered the FST as previously described in Lehmann et al., 2011. Mice were briefly placed into a clear plastic bucket, with dimensions of 20cm in diameter and 60 cm in height. The bucket was filled to 23 cm in depth with 22°C water. Mice were exposed to the test for 2 consecutive days and placed in the water for 6 minutes per session. Mice were videotaped from the side to measure floating duration. Behavior was scored using an automated Viewpoint Videotrack software package (http://www.viewpoint.fr/en/a/anxiety-and-depression).

Novelty Suppressed Feeding (NSF). Novelty suppressed feeding was performed as previously described in Lehmann et al., 2013. Mice were briefly placed into a brightly light (1100-1200 lux) arena with approximately 2 cm of wooden bedding covering the floor. Prior to testing, all mice were food restricted for 12 hours and all food was removed from the home cage. During testing, a single pellet of food was placed in the center of the arena on top of a circular white paper disk. To initiate the test, mice were placed in the corner of the arena, and a stopwatch was immediately started. Latency of time to begin feeding was recorded. Once the mouse took its first bite of the pellet, food was removed from the arena, and the mouse was placed back into its home cage. Home cage consumption was then measured as the amount of food consumed in 5 minutes, followed by a measurement of weight post-food restriction. All behavior was videotaped and later analyzed using a Kaplan-Meier survival plot for the distribution of data. We also used the Mantel-Cox log-rank test to evaluate differences between the experimental groups.

Elevated Plus Maze (EPM). The elevated plus maze was performed as previously described in Lehmann et al., 2014. Mice were briefly placed into the center of a plus-cross-shaped maze

consisting of four arms, two that are open and two that are enclosed by walls. The two arms are linked by a central platform at a height of 50 cm from the floor. Each mouse was individually placed into the center of the maze and were allowed to explore for 5 minutes. Time spent in open versus closed arms was used to measure anxiety-like behavior in male 129S6/SvEv mice. Videos were scored using behavioral tracking software (TopScan, CleverSys, Reston, VA).

Contextual Fear Conditioning (CFC). The 1-shock CFC procedure was performed as previously described in Brachman et al., 2015. Mice were briefly placed into context A and administered a 2-s shock (0.75 mA) 180 s later. Mice were then removed from the context 15 s following the termination of shock (at 197 s). For context retrieval, mice were placed back into context A for 180 s, but received no foot shocks.

References

Brachman, R.A., Lehmann, M.L., Maric, D., and Herenham, M. (2015). Lymphocytes from chronically stressed mice confer antidepressant-like effects to naïve mice. *J Neurosci* 35, 1530-8. doi: 10.1523/JNEUROSCI.2278-14.2015.

Donahue, R.J., Muschamp, H.W., Russo, S.J., Nestler, E.J., and Carlezon W.A., Jr. (2014). Effects of striatal ΔFosB overexpression and ketamine on social defeat stress-induced anhedonia in mice. *Biol Psychiatry* 6, 550-8. doi: 10.1016/j.biopsych.2013.

Lehmann, M.L. and Herkenham, M. (2011). Environmental enrichment confers stress resiliency to social defeat through an infralimbic cortex- dependent neuroanatomical pathway. J Neurosci 31, 6159-6173. doi: 10.1523/JNEUROSCI.0577-11.2011.

Lehman M.L., Mustafa, T., Eiden, A.M., Herkenham, M., and Eiden, L.E. (2013). PACAP-deficient mice show attenuated corticosterone social defeat stress. *Psychneuroendocrinology* 38, 702-15. doi: 10.1016/j.psyneuen.2012.09.006.

Lehman, M.L., Brachman, R.A., Martinowich, K., Schlosser, R.J., and Herkenham, M. (2013). Glucocorticoids orchestrate divergent effects on mood through adult neurogenesis. *J Neurosci* 33, 2961-72. doi: 10.1523/JNEUROSCI.3878-12.2013.

Supplementary Figure Legends

Figure S01. High doses of ketamine increase mitotic cell division in the hippocampus. **(A)** Timeline indicating administration of either Sal, PB, or KX. **(B)** KX significantly increased the number of $BrdU^+$ cells in the hippocampus. (n = 6 male mice per group). Error bars represent ± SEM. * $p < 0.05$, ** $p < 0.01$, *** $p < 0.001$. Sal, saline; PB, pentobarbital; KX, ketamine/ xylazine mixture; BrdU, 5-bromo-2'-deoxyuridine; Eut, euthanize.

Figure S02. The number of DCX^+ cells significantly increases along the dorsoventral axis of the HPC. **(A-B)** In the prophylactic cohort, the number of DCX^+ cells per section and the number of DCX^+ cells with tertiary dendrites per section increased along the dorsoventral axis of the DG. **(C-D)** In the antidepressant cohort, the number of DCX^+ cells and the number of DCX^+ cells with tertiary dendrites per section increased along the dorsoventral axis of the DG. (n = 6-9 male mice per group). Error bars represent ± SEM. * $p < 0.05$, ** $p < 0.01$, *** $p < 0.001$. DCX, doublecortin.

Table S01. Statistical analysis summary.
