# Supplementary figures and images for: Antidepressant but Not Prophylactic Ketamine Administration Alters Calretinin and Calbindin Expression in the Ventral Hippocampus
Source: Front Mol Neurosci. 2018 Nov 6;11:404. doi: 10.3389/fnmol.2018.00404 (PMC6232342; doi:10.3389/fnmol.2018.00404)

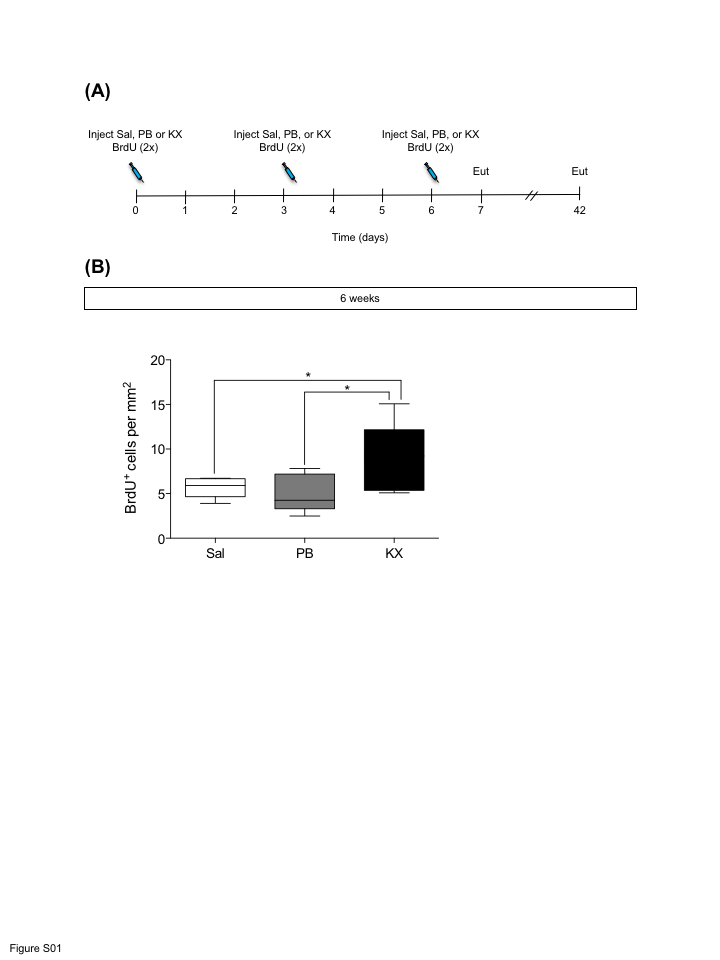

Supplement: Supplementary file 3 [file Image_1.TIFF]

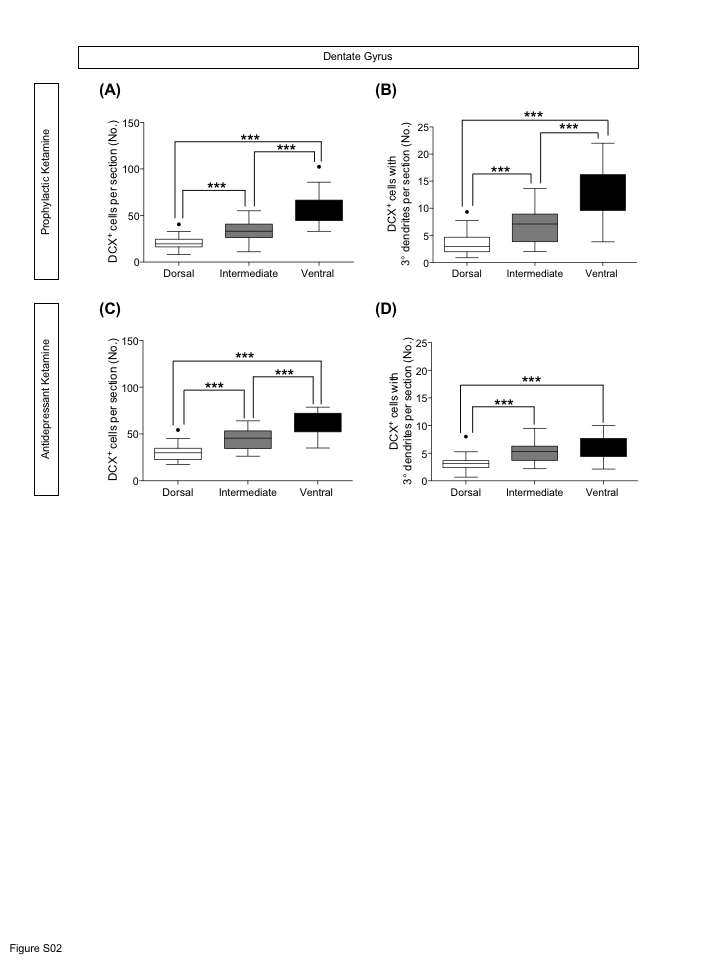

Supplement: Supplementary file 4 [file Image_2.TIFF]
